# The Allergenic Activity of Blo t 2, a *Blomia tropicalis* IgE-Binding Molecule

**DOI:** 10.3390/ijms24065543

**Published:** 2023-03-14

**Authors:** Ernesto Mondol, Karen Donado, Ronald Regino, Karen Hernandez, Dilia Mercado, Ana Carolina Mercado, Inés Benedetti, Leonardo Puerta, Josefina Zakzuk, Luis Caraballo

**Affiliations:** Institute for Immunological Research, University of Cartagena, Cartagena 130014, Colombia

**Keywords:** *Blomia tropicalis*, allergen, house dust mites, animal model, Blo t 2, IgE, humans

## Abstract

Only few allergens derived from house dust mite (HDM) species have been evaluated in terms of their potential to induce allergic inflammation. In this study, we aimed to evaluate different aspects of the allergenicity and allergenic activity of Blo t 2, a *Blomia tropicalis* allergen. Blo t 2 was produced as a recombinant protein in *Escherichia coli*. Its allergenic activity was tested in humans by skin prick test and basophil activation assays, and in mice, by passive cutaneous anaphylaxis and a model of allergic airway inflammation. Sensitization rate to Blo t 2 (54.3%) was similar to that found to Blo t 21 (57.2%) and higher than to Der p 2 (37.5%). Most Blo t 2-sensitized patients showed a low intensity response (99.5%). Blo t 2 elicited CD203c upregulation and allergen induced skin inflammation. Additionally, immunized animals produced anti-Blo t 2 IgE antibodies and passive transfer of their serum to non-immunized animals induced skin inflammation after allergen exposure. Immunized animals developed bronchial hyperreactivity and a strong inflammatory lung reaction (eosinophils and neutrophils). These results confirm the allergenic activity of Blo t 2 and supports its clinical relevance.

## 1. Introduction

*Blomia tropicalis* and *Dermatophagoides pteronyssinus* dominate the mite fauna composition of tropical dwellings [1,2,3], where they are important inducers of sensitization among asthmatic patients. Despite more than 40 IgE-binding components of these house dust mite (HDM) species being discovered, their potential to induce allergic inflammation was only explored in some of them [4]. The best characterized allergens belong to *Dermatophagoides* genus, being their group 1 and group 2 members frequent sensitizers of allergic patients. Der p 1 and Der f 1 are cysteine proteases whose enzymatic activity promote epithelium damage and type-2 inflammation. The allergenic activity of group 13 allergens, Der p 13 and Blo t 13, was also well analyzed [5]. Der p 2 is a lipid binding allergen whose biological interaction with toll like receptor 4 (TLR-4) was demonstrated to be essential for its allergenic activity. However, little is known about the allergenic activity of the orthologous molecule Blo t 2 from *B. tropicalis* far beyond its IgE binding frequency. Reginald et al. characterized the IgE reactivity of Blo t 2, a polymorphic group containing at least nine naturally occurring isoforms. These authors reported that this allergen sensitized about 40% of Singaporean allergic subjects and is present in house dust [6].

Currently, it is recognized that the allergenic activity (the capacity of inducing inflammation) of a molecule may depend on IgE-mediated reactions, but also on innate immune mechanisms [4]. Since the definition of a molecule as an “allergen” is defined by the IgE-binding capacity (which means its allergenicity), the translation into a meaningful allergic reaction is often neglected. Some allergens may be classified as clinically important because they are common sensitizers (>50%), even lacking evidence about its allergenic activity [7]. On the other hand, molecules with lower rates of IgE recognition can promote allergic inflammation and are essential during the induction process of an allergic reaction. Good examples of this are the invertebrate tropomyosins [8] and the fatty acid binding proteins [5]. Therefore, determining the allergenic activity of IgE-binding components is an important step that helps to identify clinically relevant allergens.

In this work, we aimed to evaluate different aspects of the allergenicity and allergenic activity of one isoform of Blo t 2. Our data showed that Blo t 2 was a common sensitizer in the study population, but the strength of the IgE responses was lower than other allergens of recognized allergological importance, such as Der p 2 and Blo t 21. Detected anti-Blo t 2 IgE levels represented genuine responses against this allergen since the lack of cross-reactivity with Der p 2 was confirmed. Furthermore, Blo t 2 induced allergic inflammation in both in vivo and in vitro models, which confirmed its allergenic activity, thus supporting its clinical relevance.

## 2. Results

### 2.1. Purification of Blo t 2

Blo t 2.0104 was efficiently produced a His-tagged protein with 23 extra amino acids (Supplementary Figure S1) and a theoretical molecular weight of 16.1 kDa. SDS-PAGE revealed that Blo t 2 ran as a 19 kDa band under reducing conditions and had >99% purity after affinity purification and anion exchange chromatography (Supplementary Figures S2 and S3). As observed in a Western blot analysis with anti-Hist tag antibody, Blo t 2 also migrated at ~40 kDa, suggesting dimer formation (Supplementary Figure S4).

Endotoxin concentration was high in the obtained purified fraction (>100 EU/mL). Passes through the polymyxin B column did not efficiently remove lipopolysaccharide (LPS) from the sample. A second purification method based on Triton X-114 treatment reduced LPS content to a final concentration of 6.4 EU/mL [0.03 EU/µg of protein]. As LPS binding activity was described for HDM group 2, the subsequent experiments with this protein were performed at this low, but still detectable, level of endotoxin content.

### 2.2. Specific IgE to Blo t 2 and Der p 2 Exhibit Differences

**Figure 1:**
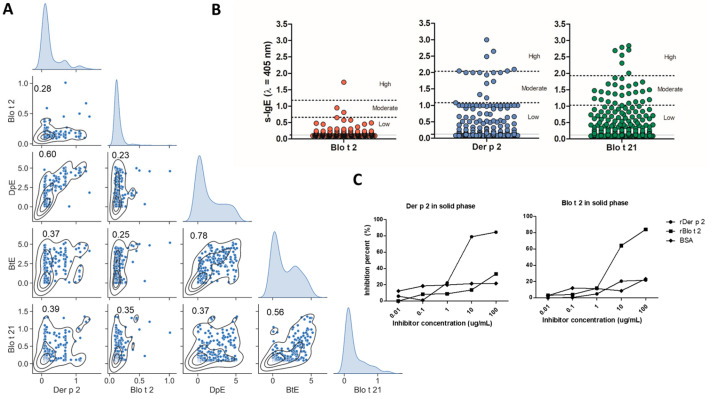
Features of the IgE response to Group 2 HDM allergens in asthmatic patients. (**A**) Scatterplot matrix of log transformed specific IgE to Blo t 2, Der p 2, Blo t 21, *B. tropicalis* and *D. pteronyssinus* extracts (*n* = 268). Kernel density estimation (KDE) contours are depicted inside each bivariate plot (Rho values from Spearman test are indicated inside each plot). In the diagonal of the matrix, KDE curves depicting univariate distributions of IgE measurements (**B**) Strip plots of specific-IgE t(s-IgE) to Blo t 2 (red), Der p 2 (blue) and Blo t 21 (green). Solid gray line in each graph represents the cut-off to define a positive test (O.D. = 0.12 for the three allergens). Dotted lines set limits for an arbitrary intensity based-classification. (**C**) ELISA inhibition curves between Blo t 2 and Der p 2 using serum pools of double-sensitized asthmatic patients.

Sensitization to the group 2 HDM allergens, Blo t 2 and Der p 2, was evaluated in 286 patients from the asthma severity cohort. The prevalence of sensitization to Blo t 2.0101 (54.3%) was similar to that found to Blo t 21 (57.2%) and higher than to Der p 2 (37.5%). As observed in the univariate kernel density estimation (KDE) curves, Blo t 2 sIgE values were rather low in most patients, in contrast to Der p 2, Blo t 21 and HDM extracts, which displayed bimodal distributions with stronger IgE responses among the positives (Figure 1A). Moderate/high specific IgE values to Blo t 2, Blo t 21 and Der p 2 were observed in 0.5%, 20% and 15% of sensitized patients, respectively (Figure 1B). Correlation between Der p 2-sIgE and Blo t 2-sIgE was low but significant (rho: 0.28, *p* < 0.01). Inhibition assays also showed a low capacity of Blo t 2 to inhibit Der p 2 and vice versa (Figure 1C).

Specific IgE to *B. tropicalis* extract correlated better with Blo t 21 s-IgE (rho: 0.56) than with Blo t 2 s-IgE (rho: 0.25), in contrast to that observed for Der p 2, which showed a higher correlation with sIgE to Dp extract (Figure 1A).

### 2.3. rBlo t 2 Degranulates Human Basophils and Mast Cells

**Figure 2:**
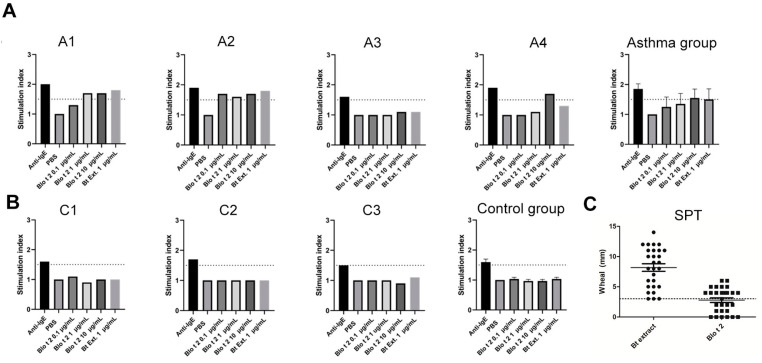
CD203c-based basophil activation test and SPT results with Blo t 2. Stimulation index of basophils stimulated with Anti-IgE as positive control, *B. tropicalis* extract (1 µg/mL) and rBlo t 2 at different concentrations (0.1, 1 and 10 µg/mL) in Blo t 2-sensitized subjects. Dotted line represents the cutoff value (SI: 1.5) to define a test as positive (**A**) individual results obtained from 4 asthmatic patients and summary statistics for the group are shown. (**B**) Plots for 3 sensitized participants without clinical allergy. Summary statistics for the control group are shown in the right. (**C**) Comparison between the diameter of the wheal produced by extract of *B. tropicalis* and rBlo t 2 in the SPT performed to 28 participants. Dotted line corresponds to the cut-off point of 3 mm to define a test as positive.

Three out of four serum samples from Blo t 2-sensitized asthmatic patients activated human basophils (Figure 2A). In contrast, none of the 4 human samples from sensitized Blo t 2 non-allergic participants induced degranulation in the passive CD203c-based basophil activation assay (Figure 2B). Fifteen out of twenty-eight *B. tropicalis* patients (53%) with respiratory allergy showed a positive skin prick test (SPT) to rBlo t 2. Average wheal size among positives was 4.4 mm. Mean wheal diameter induced by Blo t 2 was significantly lower than that induced by *B. tropicalis* extract (Figure 2C).

### 2.4. rBlo t 2 Induces Inflammation in a Murine Model of Allergic Asthma

**Figure 3:**
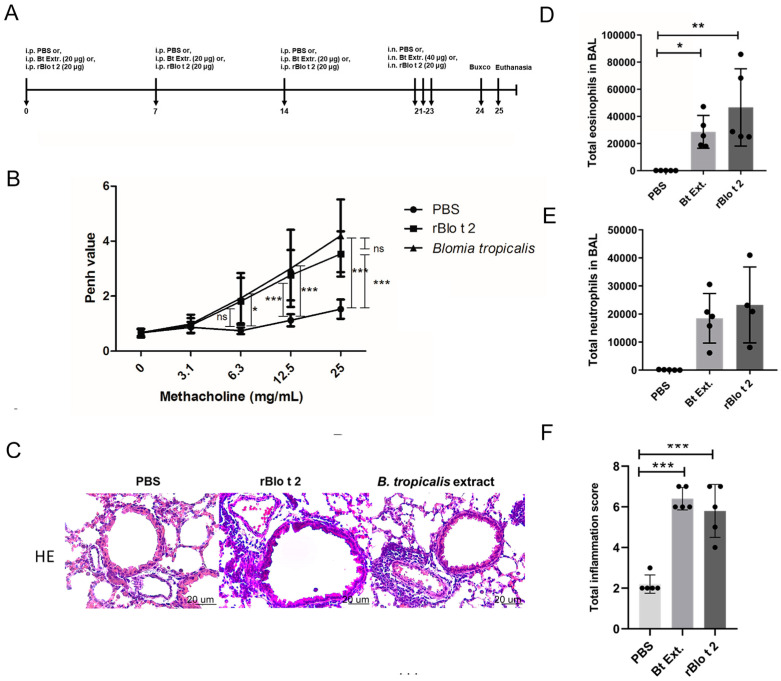
Mouse model of Blo t 2-induced airway inflammation. (**A**) Sensitization and challenge protocol for the murine model of allergic asthma. Mice were sensitized via intraperitoneal (i.p.) injections on days 0, 7 and 14, followed by daily intranasal (i.n.) challenges on days 21, 22 and 23. Airway hyperreactivity was monitored by whole body plethysmography on day 24. Inflammatory cell count of the bronchoalveolar lavage samples. (**B**) Pehn values obtained in the WBP measurements (**C**) representative images of stained hematoxylin-eosin (HE) lung sections. Absolute counts of eosinophils (**D**) and neutrophils (**E**) in the bronchoalveolar lavage of mice. The data are reported as means ± SE (*n* = 5 per group). (**F**) Summary statistics for inflammation score in the lung histology analysis. Between-group comparisons were performed using the one-way ANOVA test and Dunnett’s test, as post hoc analysis (ns: not significant * *p* < 0.05 ** *p* < 0.01 *** *p* < 0.001).

To evaluate the capacity of Blo t 2 to induce lung inflammation, mice were immunized with this allergen via i.p. and further challenged via nasal exposure (Figure 3A). The complete *B. tropicalis* extract was used as a positive control. In the whole body plethysmography (WBP), mice receiving rBlo t 2 or *B. tropicalis* showed higher Penh values after challenge with methacholine (Mch) than those receiving PBS. *B. tropicalis* immunized animals started to show significant differences compared to the control group from a lower Mch dose (6.3, 12.5 and 25 mg/mL) than rBlo t 2 (12.5 and 25 mg/mL); however, Pehn values were not different between rBlo t 2 and *B. tropicalis* at any dose (Figure 3B). Lung tissue sections showed signs of inflammatory infiltration in the *B. tropicalis* and rBlo t 2-exposed groups (Figure 3C,F). High inflammatory scores were detected in both *B. tropicalis* and Blo t 2-immunized groups, where ~50% of infiltrating cells were identified as neutrophils. The induced inflammatory responses were also reflected in BAL cell counts; both allergen stimuli induced local mixed responses characterized by a high number of eosinophils and neutrophils (Figure 3D,E), as detected by flow cytometry identification using lineage cell markers (Supplementary Figure S5).

**Figure 4:**
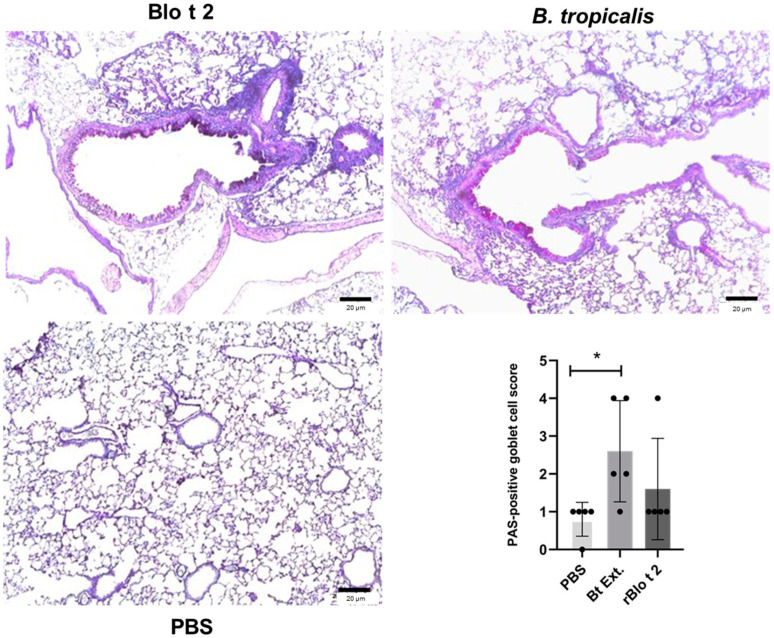
Histological analysis of mucus production. Representative images of periodic acid-Schiff (PAS)-stained lung tissue sections from each group are shown. Summary statistics for all individuals are presented in the bar plot. Error bars represent the standard error of the mean (S.E.M). Comparisons were made using the one-way ANOVA test and Dunnett’s test, as post hoc analysis. * *p* < 0.05.

In contrast to the complete HDM extract, rBlo t 2 did not induce significant goblet cell hyperplasia, as evidenced by periodic acid-Schiff staining (Figure 4).

**Figure 5:**
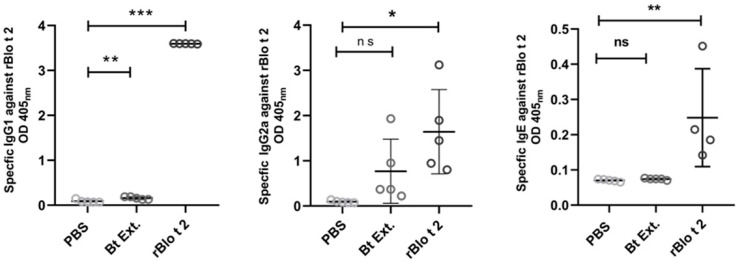
Serum antibody responses in mice. rBlo t 2-specific IgG1, IgG2a and IgE were measured in sera of mice sensitized with rBlo t 2, *B. tropicalis* extract (Bt Ext.), and PBS. Data are reported as individual OD. Error bars represent the mean value of each group and their standard error. The comparison was performed using the one-way ANOVA test and Dunnett’s test, as post hoc analysis. * *p* < 0.05 ** *p* < 0.01 *** *p* < 0.001, ns: not significant.

Furthermore, rBlo t 2 administration induced allergen specific IgE, IgG1 and IgG2a antibodies compared to the control group (Figure 5). Mice immunized with *B. tropicalis* also developed anti-Blo t 2 antibodies of IgG1 and IgG2a isotypes but failed to induce detectable levels of anti-Blo t 2 IgE.

### 2.5. rBlo t 2 Induces Passive Cutaneous Anaphylaxis

**Figure 6:**
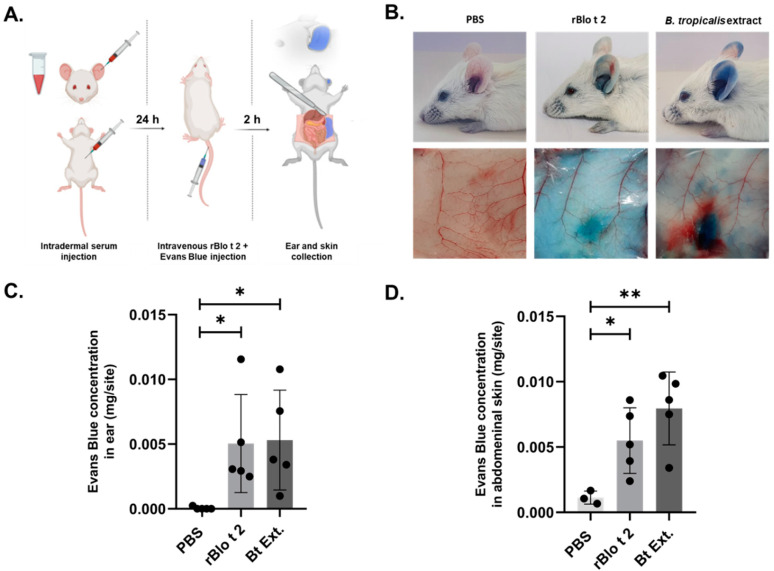
Allergenic activity of rBlo t 2 evaluated in a passive cutaneous anaphylaxis model. (**A**) The PCA protocol was performed in 3 groups of BALB/c mice. (**B**) Representative images of PCA reactions showing extravasated Evans Blue on the ear and abdominal skin of *B. tropicalis* extract and rBlo t 2 groups. Quantification of Evans Blue extracted after ACP reaction in (**C**) the ear and (**D**) abdominal skin, respectively. The data are reported as means ± standard errors of Evans Blue concentration expressed in mg/site (*n* = 3–5 per group). The comparison was performed using the one-way ANOVA test and Dunnett’s test, as post hoc analysis. * *p* <0.05 ** *p* <0.01.

Mouse sera from the airway inflammation model were further evaluated in passive cutaneous anaphylaxis (PCA) assay. As observed in Figure 6, Evans blue extravasation was observed in mice injected with *B. tropicalis* extract and rBlo t 2 group-derived sera; meanwhile, sera from the PBS-exposed group did not induce a positive reaction. Measurements from the abdominal area indicated a more intense reaction induced by the *B. tropicalis* complete extract.

## 3. Discussion

Although *B. tropicalis* is one of the most important causes of allergy in tropical and sub-tropical zones [9,10,11], the allergenic potential of the individual molecules mediating its strong capacity to induce clinical symptoms were not completely studied. Up to 14 allergens were registered [10], but for only few of them, there was a detailed evaluation of each allergen’s capacity to induce IgE production, tissue inflammation and IgE-mediated cell degranulation, which are important criteria for defining the allergenic activity [4]. In this report, we focused on the allergenic activity of the Blo t 2.0101, a group 2 isoform that sensitizes 37–54% of allergic patients from different populations living in tropical countries [12,13,14]. As published by Reginald et al., *B. tropicalis* contain at least nine different isoforms, where the isoform 1 seems to be the most abundant when analyzed by cDNA clone’s frequency and, also, at the protein level within the extract [6,15]. Then, our results were expected to be highly representative of the group 2 from *B. tropicalis*, but it was possible that other isoforms had different immunological behavior and contributions to the allergenic repertoire of the mite. We showed that Blo t 2 induces IgE production by itself, and it is effective in inducing biological responses associated with IgE-mediated degranulation, such as CD203c upregulation as well as allergen-induced skin inflammation as detected by the skin prick test. Additionally, immunized animals produced anti-Blo t 2 IgE antibodies and passive transfer of their serum to non-immunized animals induced extravasation reactions after allergen exposure, which also supports the ability of Blo t 2 to produce an effector allergic reaction.

Others analyzed the prevalence of sensitization to Blo t 2, reporting similar rates to ours [12,13,14]. Since the frequency of positive IgE responses to Blo t 2 were as high as those reported for other clinically relevant allergens such as Blo t 5 and Blo t 21, and monosensitization to Blo t 2 is common [16], it was proposed as a component that improves the diagnostic performance of molecular panels to detect sensitization to *B. tropicalis*. Classification of specific IgE values into intensity ranges indicated that most positive patients had low intensity responses, as also observed by others [6,16]. Nevertheless, we demonstrated by different methods that these anti-Blo t 2 IgE antibodies were able to induce allergic reactions. Additionally, it was significant that the detected Blo t 2 antibodies were from primary sensitization, and not of cross-reactivity with Der p 2, with which the molecule shares >50% of sequence similarity.

When analyzing the correlation of IgE responses, we observed low correlation between Blo t 2 and Der p 2 that coincided with low inhibition of IgE binding between Blo t 2 and Der p 2. In contrast, *B. tropicalis* and *D. pteronyssinus* were moderately correlated, an expected finding [17], due to the co-exposition and presence of highly cross-reactive allergens such as group 10 [18,19]. We also observed that compared with Blo t 2, Der p 2 and Blo t 21 were more correlated with the IgE response towards the extract species to which they belong. Due to its high abundance in the extract (~50% in some preparations), we expected to find a higher correlation between the specific IgE responses; however, Der p 2 and Blo t 21 were superior in representing the IgE response to the complete extract in spite of their lower relative abundance [15,20]. We have no accurate explanation for this, but some comments can be made. The relationship between protein abundance and its allergenic potential is not clear, but similar findings support that exposure to high levels of HDM allergens are related inversely with IgE responses [21,22]. When Reginald et al. measured group 2 HDM allergen levels in the house dust, they found that Blo t 2 was more abundant than Der f 2, but the latter induced stronger IgE responses [6]. Interestingly, we previously found that the intensity of the IgE response to Blo t 5 was inversely related to the allergen abundance in house dust of children living in Cartagena, Colombia [22].

Blo t 2 was capable of generating airway inflammation, reflected by the bronchial hyperresponsiveness observed in exposed mice. The allergen provoked a strong neutrophilic bronchial infiltrate, which suggested that it also activated other inflammatory pathways as well as Th2-mediated responses. A similar neutrophilic effect was described earlier in Der f 2-exposed mice [23]. Additionally, it was reported that Der f 2 and Der p 2 bind LPS and that this biological activity may promote allergenicity through a TLR4-dependent mechanism [24]. Although we did not formally evaluate the LPS binding activity of Blo t 2, it was observed that LPS derived from the bacterial expression system could not be completely removed from protein solution using polymyxin B column. The observation suggests that Blo t 2 has an appreciable affinity to this ligand. Endotoxin airway exposure may promote bronchial hyperresponsiveness and activation of innate immune signaling pathways that may lead to neutrophilia and exacerbate asthmatic responses. Der p 2 and LPS showed synergistic effects on IL-6 and IL-8 production by BEAS2B lung cell lines [25] and MD2 upregulation on whole blood cells [26]. Thus, it is possible that Th1 and innate immunity pathways were involved in our Blo t 2-induced experimental asthma model, possibly associated with its potential LPS binding activity. In fact, it was reported that Blo t 7 and other HDM allergens [27,28,29] can induce the production of IL-8, a neutrophil recruiter and activator, through the TLR2 receptor, then it should be necessary to explore if Blo t 2 can use this or other innate pathways. The production of anti-Blo t 2 may also have an effect on the inflammatory response associated with eosinophilia. Although it is possible that alum may have induced non-specifically the production of IgE, we must take into consideration that not all proteins induced IgE when administered to mice in the presence of alum, as we previously observed with a parasite immunomodulator from *Ascaris lumbricoides* [30]. In fact, it was demonstrated that alum may have an enhancement effect on other immunoglobulin isotypes, but not IgE [31].

Interestingly, the presence of neutrophils in lung inflammatory infiltrates was also found after exposure to the complete *B. tropicalis* extract. Carvalho et had previously noticed that the inflammatory lung infiltrate elicited by *B. tropicalis* was rich in neutrophils, in contrast to *D. pteronyssinus* that induced only eosinophil-rich lung infiltrates in exposed mice [32]. It is possible that the behavior of *B. tropicalis* extract may be due to its high group 2 content, which may be as high as 50% of protein content. Although the IgE binding potency of *D. pteronyssinus* was highly determined by Der p 2, the absolute protein quantities are lower. In a proteomic analysis of *D. pteronyssinus* extracts, Batard et al. found that Der p 2 was abundant, but it was in the top 42 position after several other structural proteins and known allergens such as group 13, 1 and 10 [20].

Several limitations of our studies must be stated. The strength of specific IgE to the different allergens could not be compared because they were measured in independent ELISA assays without calibrators allowing external comparisons. Although the presence of endotoxin in the recombinant allergen may enhance basophil activation, we consider this effect may be low since basophil reactivity to endotoxin is low; other experiments showed that only at high concentrations (>1000 EU/mL), LPS may induce cell activation [33]. Additionally, free endotoxins may have induced neutrophilia and not bound LPS to Blo t 2. Lipid binding activity of Blo t 2 must be assessed objectively. On the other hand, Reginald et al. previously showed that Blo t 2 can be efficiently produced in a prokaryotic system, and a circular dichroism revealed that it had the appropriate secondary structure [6]. However, to increase protein yield, NLS, a mild detergent with few interferences on biological activity of recombinant proteins was used [34,35]. A more recent publication showed that this detergent may affect protein folding of insoluble proteins [36], then, the final impact on Blo t 2 is unknown. Despite demonstrating that rBlo t 2 is biologically actively inducing allergic reactions and also similar rates of IgE binding to other similar studies [6,16], physico-chemical characterization of rBlo t 2 will confirm if the recombinant has the optimum folding to represent its natural counterpart. Additionally, the animal model must be replicated to validate our findings.

In summary, our results show that Blo t 2 is a common sensitizer with confirmed allergenic activity as indicated by human SPTs and BAT assays, and a model of experimental asthma and PCA in mice. This is an important step in the general task of defining the potential clinical impact of HDM IgE-binding molecules.

## 4. Materials and Methods

### 4.1. Recombinant Allergen Production

The gene sequence of Blo t 2 isoform 1 was obtained from GenBank (ABG76185.1), codon-optimized and synthetized without the signal peptide (Genscript, Piscataway, NJ, USA) for further cloning into the pET45b+ vector. *Escherichia coli* (DE3) Origami cells were transformed with the recombinant plasmid by electroporation following manufacturer’s instructions (Bio-Rad, Hercules, CA, USA). Protein expression was induced with 0.1 mM IPTG. For lysate preparation, cells were resuspended first in 100 mM NaH_2_PO_4_ 300 mM NaCl and sonicated for 5 cycles of 20 s in a Sonic Dismembrator 705 (Fisher Scientific, Hampton, NH, USA) and then, it was homogenized with 0.2% n-lauroylsarcosine (NLS) buffer during 24 h incubation. For purification, the bacterial lysate was passed through a Ni-NTA column (Qiagen, Hilden, Germany) and eluted with 250 mM imidazole, 50 mM NaH_2_PO_4_, 300 mM NaCl (pH 8.0) for elution (Supplementary Figure S2). Then, a second purification step was carried out using anion exchange chromatography in an UNO Q1 column (Cat. 720-0001, Bio-Rad, Hercules, CA, USA) adapted to a fast performance liquid chromatography (FPLC) BiologicDuoFlow^TM^ system (Bio-Rad, Hercules, CA, USA). The column was equilibrated with 20 mM Tris-HCl pH 7.6 and rBlo t 2 was purified using a step gradient of 20 mM Tris-HCl pH 7.6 and 1M NaCl. Purified rBlo t 2 was dialyzed against PBS (pH 7.3) using membranes with a molecular weight cut-off of 6000–8000 Daltons (Spectra/Por™, Spectrum Lab, cat. 08670C). The electrophoretic behavior of Blo t 2 was observed in SDS-PAGE using reducing (5% 2-mercapto ethanol) and non-reducing conditions. rBlo t 2 was also tested with an anti-His-tag antibody (Genscript, Cat. Number A00612, Piscataway, NJ, USA). For this, we electro-transferred Blo t 2 to a nitrocellulose membrane (180 mA for 90 min). Then, membrane was blocked with 5% defatted milk, washed with 0.1% Tween 20 PBS (5X) and then incubated for 1 h with the anti-His-tag antibody at 1:100.000 dilution. For development of the chemiluminescence reaction, the Super-Signal West Femto (Thermo Scientific, Waltham, MA, USA) reagents were used. Signal was captured by a CCD-camera (G:Box Syngene, Cambridge, UK).

For endotoxin removal, rBlo t 2 was passed through a polymyxin B column (Genscript, Piscataway, USA) for endotoxin removal without successful results. A second protocol using 1% Triton X-114 was used following a protocol described elsewhere [37]. Final LPS concentration (6.4 EU/mL, 0.029 EU/µg of rBlo t 2) was quantified by a ToxinSensor Chromogenic LAL assay (GenScript, Piscataway, NJ, USA).

rDer p 2 coding sequence (P49278) was also codon-optimized, commercially synthesized and subcloned into pET45b+. Protein expression was induced under standard conditions as described above. For protein purification, bacterial pellets were lysed under standard condition following the QiaExpressionist manufacturer instructions and purified also under native conditions accordingly.

### 4.2. Human Samples

Human serum samples were obtained from a previous study which recruited asthmatic patients living in Cartagena, Colombia, during 2014. Samples were kept under −80 °C from the time of collection. In total, 268 serum samples were analyzed in terms of sensitization to Blo t 2 and Der p 2. Since IgE determinations to Blo t 21 were also available from these patients and data was used for comparisons of the strength of the IgE response.

### 4.3. Assessment of Sensitization

Specific IgE antibody levels to the house dust mites *D. pteronyssinus* and *B. tropicalis* were measured using the ImmunoCAP system as previously described [38]. Specific serum IgE levels to the recombinant allergens were detected in duplicate by indirect ELISA using an in-house protocol [39]. Threshold for positivity was set using the mean of 5 negative controls plus three standard deviations (OD: 0.12). For each allergen, positive tests were classified into low, moderate, or high intensity based on their obtained OD values. Equal width ranges for each intensity category were calculated [width = (maximum OD value − cut-off value)/3] and this value was added to the maximum limit value of the precedent category (i.e., upper limit of low class = ELISA cut-off + width).

### 4.4. ELISA Inhibition

To study cross-reactivity between Der p 2 and Blo t 2, ELISA inhibition was carried out according to a similar protocol described earlier (20). Results are shown as percentage of inhibition, calculated as follows = OD without inhibitor − OD with inhibitor/OD without inhibitor × 100. Bovine serum albumin (BSA) was used as non-related inhibitor.

### 4.5. Passive Basophil Activation Test

Serum samples from seven donors were used to evaluate basophil activation in cells obtained from a non-allergic donor. As we had previously observed in another study that non-allergic participants (Zakzuk et al., accepted for publication 16 January 2022, https://doi.org/10.18176/jiaci.0892) may be sensitized to Blo t 2, we evaluated if the presence of this antibody response translated into meaningful allergic reactions. Thus, basophil activation tests were carried out in four asthmatic patients and three non-allergic subjects (without any clinical manifestation of allergy). Sex, age and IgE data of serum samples are shown in Table S1. Ficoll (Histopaque^®^ Sigma-Aldrich, cat.1077, St. Louis, MO, USA) was used to separate peripheral blood mononuclear cells (PBMCs) and basophils from a healthy donor blood sample according to a standard protocol. These cells were filtered and washed three times with a 2 mM PBS/EDTA solution. To remove receptor-bound IgE, cells were suspended in 3 mL of lactic acid solution (13.4 mM lactate, 50 mM KCl and 140 mM NaCl) and incubated for 1 min on ice. To stop the reaction, the cell suspension was neutralized by adding 7 mL of RPMI-1640 medium supplemented with 0.5% BSA and 12 mM Tris–HCl (pH 8.0). Cells were incubated for 1 h at 37 °C with sera of 10 asthmatic patients and 6 controls. After washing with PBS, cells were incubated with rBlo t 2 at concentrations of 0.1, 1 and 10 μg/mL, *B. tropicalis* extract at 1 μg/mL, PBS as the negative control, and anti-IgE at 10 μg/mL as the positive control. The Allergenicity Kit (Beckman Coulter, Fullerton, CA, USA) was used to quantify basophil CD203c expression. Basophils were identified as SSC^low^, CD3^neg^, CRTH2^pos^ events. Flow cytometric analysis was performed on a FACS-ARIA III cytometer (BD, Franklin Lakes, NJ, USA). Results were expressed as the stimulation index (SI), the ratio of the median fluorescence intensities (MFI) after allergen stimulation/basal activation. A cut-off value of 1.5 SI was used to define a test as positive.

### 4.6. Skin Prick Test (SPT)

The SPT was performed using a panel of allergens including rBlo t 2 at 25 ng/mL and *B. tropicalis* extract, histamine phosphate solution (positive control) and diluent (negative control). The concentration of the recombinant protein for SPT was chosen after titration. Wheal size (diameter in millimeters) was measured directly on the skin. A wheal of 3 mm greater than that of the negative control was considered positive (19).

The tests were carried out by trained medical personnel on 28 volunteers with a medical diagnosis of allergic rhinitis and/or asthma and reported symptoms after house dust exposure.

### 4.7. Model of Allergic Airway Inflammation

BALB/c mice were obtained from the National Institute of Health (Bogotá, Colombia). All mice were females and used at the age of 6–8 weeks. Mice were housed under standard conditions in the animal facilities at the University of Cartagena and were climate controlled and exposed to a 12 h/12 h light/dark cycle. Animals received a standard pellet diet and drinking water ad libitum. Animal experiments were performed in accordance with institutional protocols and international regulations and were approved by the Ethical Committee of the University of Cartagena. Protocols were described in previous studies from our research group. Mice were sensitized three times (days 0, 7 and 14) via i.p. injections with 150 µL of a suspension containing 20 μg of rBlo t 2 or 20 μg of *B. tropicalis* whole extract (positive control) adsorbed with 2 mg of alum (Imject^®^ Alum, Thermo Fisher Scientific, Waltham, MA, USA) at 1:2 ratio. Subsequently, on days 21, 22 and 23 animals were challenged intranasally with 20 μg of rBlo t 2 or 40 μg of *B. tropicalis* extract, respectively. Naive control mice were injected with alum in PBS and challenged using PBS (25 µL per nostril).

### 4.8. Measurement of Airway Hyperreactivity

Lung function was assessed on day 24 by methacholine-induced airflow obstruction, using a whole-body plethysmograph (Buxco Electronics, USA). Methacholine (Sigma-Aldrich, cat. A2251-25G) was diluted in water at different concentrations (25, 12.5, 6.25 and 3.12 mg/mL) and administered in aerosol (50 μL per chamber for 90 s). Changes in Penh (enhanced pause) values were used as an indirect measure of airway hyper-reactivity.

### 4.9. Histology

Mice were sacrificed by using a lethal dose of anesthesia (Euthanex^®^) 24 h after plethysmography. The left lung was extracted and immersed in 10% neutral-buffered formalin. The lung was embedded in paraffin and then cut for hematoxylin-eosin (HE) and periodic acid-Schiff (PAS) staining. Lung inflammation and mucus production was evaluated by a pathologist blinded for group assignments. To determine the severity of inflammatory cell infiltration, peribronchial, and perivascular inflammatory cell counts were performed blind based on a modification of the 5-point scoring system described by Myou et al. [40].

### 4.10. Bronchoalveolar Lavage (BAL)

BAL was harvested by flushing the lung airways via the trachea (2X) with 1 mL of ice-cold PBS containing a protease inhibitor cocktail (Roche, Germany). After centrifugation for 5 min at 1.500 rpm at 4 °C, supernatants were collected and stored at −80 °C. The cell pellet was resuspended in 1 mL of PBS and mixed with 1 mL of 1X lysis buffer, it was left in incubation for 5 min on ice and then centrifuged at 1.500 rpm for 5 min. Cells were resuspended in 160 μL of Stain buffer (PBS 0.5% BSA), and 40 μL of this suspension was mixed with 10 μL of the monoclonal antibodies cocktail (V450-CD45, PECy7-CD3e, FITC-F4/80, PE-CD11b, PerCPefluor-CD170 (SiglecF), APC-Ly6G) (Table S2) or 10 μL of the isotype control cocktail to define the different cell populations. Stain reaction was incubated for 10 min at 4 °C in the dark and washed by centrifugation with 500 µL of Stain Buffer for 7 min at 300 G. Finally, cells were resuspended in Fixation Buffer (PBS + 0.1% formaldehyde) and the different cell populations were analyzed by flow cytometry using the FACS-ARIA III system (BD, Franklin Lakes, NJ, USA).

### 4.11. Mouse Antibody Analyses

Specific IgE, IgG1 and IgG2a levels were determined by ELISA. Microtiter plates were coated with 0.25 μg of rBlo t 2 in 50 μL of sodium carbonate/bicarbonate buffer (pH 9.6) and incubated overnight at 4 °C. Wells were then blocked with PBS 1% BSA. Then, 50 μL of plasma samples diluted and 1:500 (IgG1 ELISA), 1:250 (IgG2a ELISA), or 1:6 (IgE ELISA) were added and incubated for 2 h at 37 °C in a humid chamber. After 5 washes, wells were incubated with biotin-labeled anti-mouse IgE, IgG1 or IgG2a (diluted 1:1.000 in blocking buffer) for 1 h at RT. After 5 washes, alkaline-phosphatase streptavidin (diluted 1:2.000 in 50 mM Tris, 1% BSA and 1 mM MgCl_2_ pH 8.0) was added to the wells and incubated for 1 h. P-nitrophenylphosphate at 1 mg/mL dissolved in 10% diethanolamine 0.5 mM MgCl2 was used as substrate solution. After 60 min of incubation, the reaction was stopped with 3N NaOH. Optical densities were obtained by reading the plates at 405 nm in a spectrophotometer. To increase the sensitivity of IgE ELISA, IgG immunoglobulin was depleted in plasma samples by incubation with protein G sepharose (Thermo Fisher Scientific, Waltham, MA, USA).

### 4.12. Passive Cutaneous Anaphylaxis (PCA) Model

The PCA model was performed as described previously (17). In brief, naive 6 to 8-week-old female BALB/c mice were divided into three different groups: rBlo t 2, *B. tropicalis* extract as the positive control, and PBS as the negative control. They were injected intradermally with 10 µL of plasma from immunized mice into the left ear and 50 µL into abdominal skin. After 24 h, each mouse was injected via the tail vein with 200 µL containing 25 µg of rBlo t 2, 40 µg of *B. tropicalis* extract or PBS, plus 0.5% Evans Blue (Panreac, Ref. 255486.1606) dissolved in PBS. Two hours later, the animals were sacrificed by a lethal dose of Euthanex^®^. The left ear and one square centimeter of the abdominal skin were collected in each mouse and incubated in 700 μL of formamide at 37 °C for 72 h. Subsequently, the amount of dye extravasated was quantified using an Evans Blue concentration curve and measuring samples at 620 nm in a spectrophotometer (Multiskan™ Thermo Fisher Scientific, Waltham, MA, USA). The results were expressed in mg of dye/site (mg/site).

### 4.13. Statistical Analysis

Correlation plots depicting the distribution of IgE data obtained from the asthmatic group were generated with seaborn version 0.11.2. in Python 3.9 (64-bit). Since data were not normally distributed, the Spearman test was applied to explore correlations. Bivariate KDE plots were generated. In this, x, y observations were smoothed with a 2D Gaussian, generating density contours.

For comparison of means of more than two groups, one-way ANOVA and Dunnett’s multiple comparison test, as post hoc analyses, were used. Differences between proportions were analyzed by Pearson’s chi-squared test. GraphPad Prism version 5.01 for Windows (GraphPad Software, San Diego, CA, USA) was used for these analyses.

## Data Availability

Datasets containing information about IgE values in the analyzed sample study are available as a supplementary file.

## Supplementary Materials

The following supporting information can be downloaded at: https://www.mdpi.com/article/10.3390/ijms24065543/s1.
