# Supplementary material for: The Allergenic Activity of Blo t 2, a Blomia tropicalis IgE-Binding Molecule
Source: Int J Mol Sci. 2023 Mar 14;24(6):5543. doi: 10.3390/ijms24065543 (PMC10053487; doi:10.3390/ijms24065543)
Supplement: Supplementary file 1 [file ijms-24-05543-s001.zip › ijms-2161947-supplementary.pdf]

A

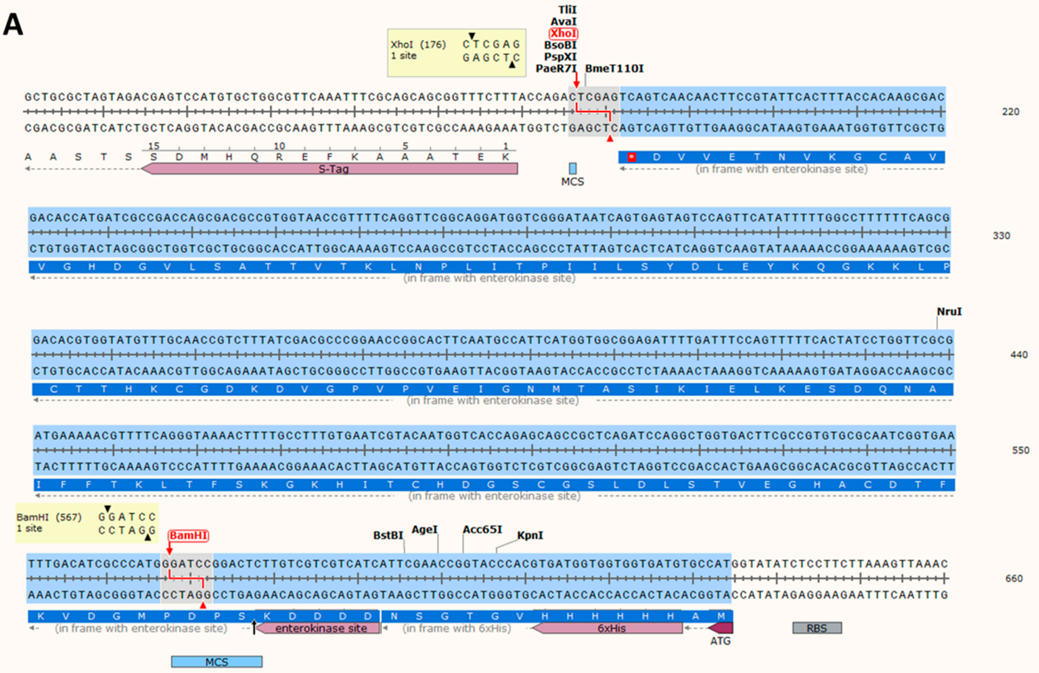

B

```
         10         20         30         40         50         60
MAHHHHHHVG TGSNDDDDKS PDPMGDVKFT DCAHGEVTSL DLSGCSGDHC TIHKGKSFTL

         70         80         90        100        110        120
KTFFIANQDS EKLEIKISAT MNGIEVPVPG VDKDGCKHTT CPLKKGQKYE LDYSLIIPTI

        130        140        150
LPNLKTVTTA SLVGDHGVVA CGKVNTEVVD
```

Supplementary Figure S1. Blo t 2 sequence. (**A**) Plasmid map depicting the nucleotide sequence of Blo t 2 when inserted into the BamHI/XhoI site of pET45b+. (**B**) The complete amino acid sequence of recombinant Blo t 2 is shown. Extra residues added after insertion in the recombinant plasmid is highlighted in cyan. The original peptide signal of Blo t 2 was removed.

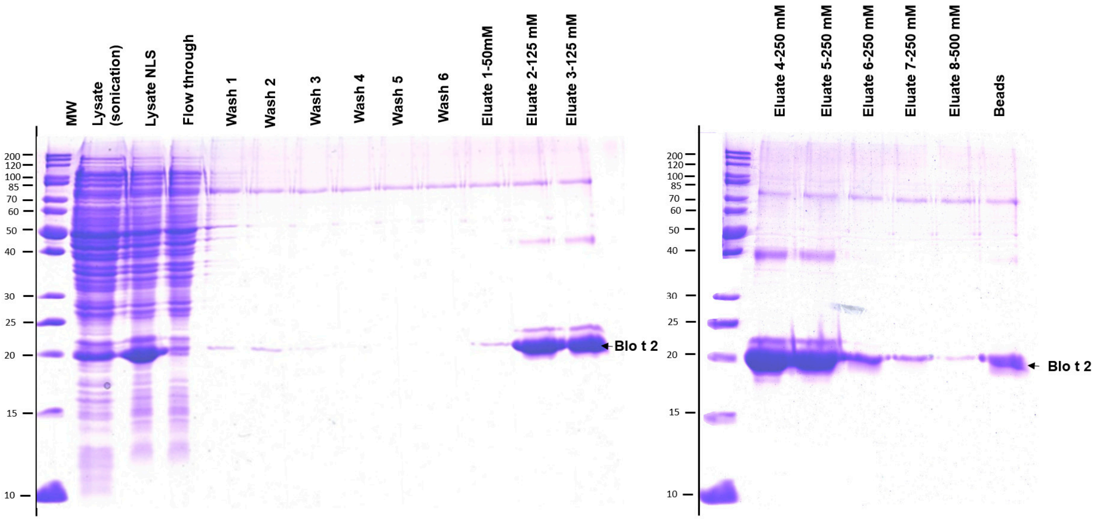

Supplementary Figure S2. Lysis and purification of Blo t 2. The pellet of Blo t 2 transformed bacteria was lysed in native lysis buffer and sonicated. In the second lane, it is observed that protein is recovered from the soluble fraction of the lysate. 0.2% NLS was added to the lysate and greater protein quantity was recovered (third lane).

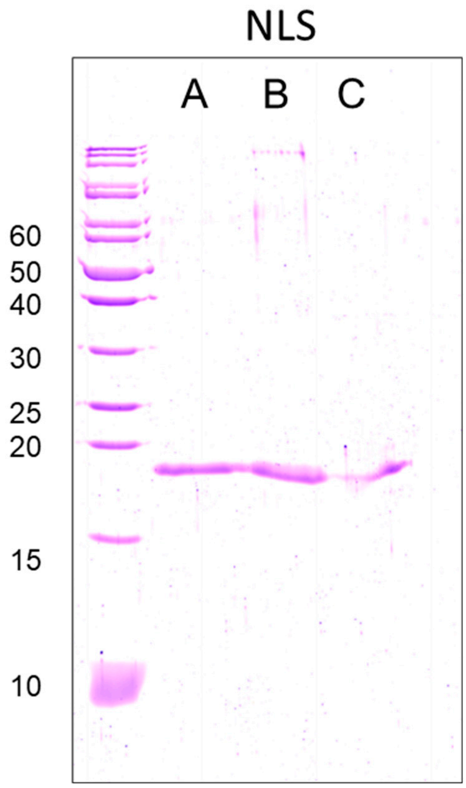

Supplementary Figure S3. SDS-PAGE of rBlo t 2. rBlo t 2 was run in a SDS-PAGE under reducing conditions and heat treatment (5% β-mercaptoethanol, Lane A) and non-reducing conditions with or without heat treatment at 95 °C for 5 minutes (Lane B and C).

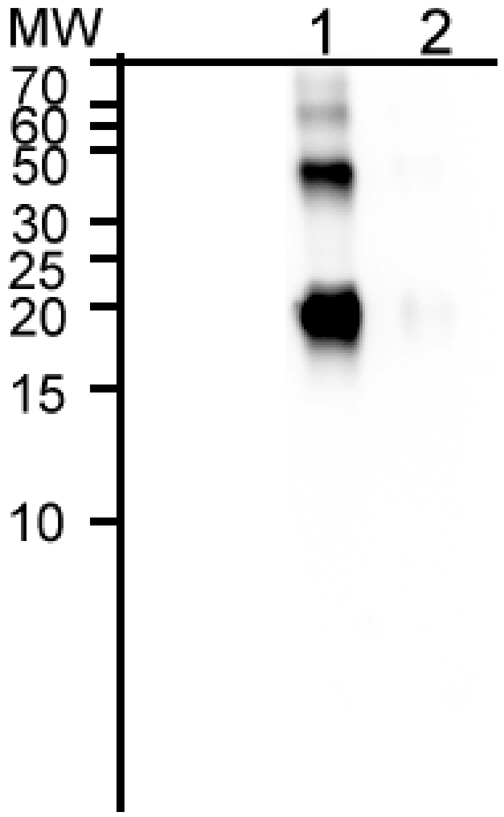

Supplementary Figure S4. Western Blot with an anti-His tag antibodies reveal dimer formation. rBlo t 2 was electro-transferred to a nitrocellulose membrane and incubated with anti-His tag antibodies (Lane 1) or blocking buffer (Lane 2). Two main bands (~20 and ~40 kDa band) are observed.

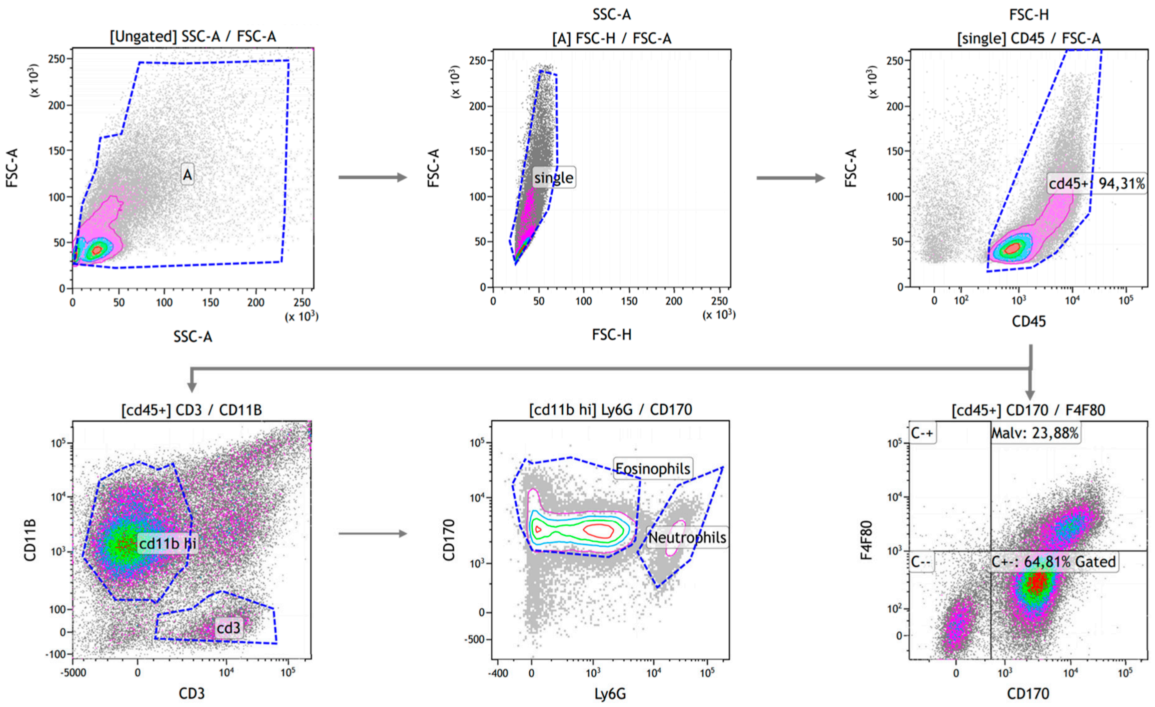

Supplementary Figure S5. Gating strategy for identification of inflammatory cells in mouse bronchoalveolar lavage. Representative flow cytometry plots from one mouse are shown. Cells were gated based on forward and side scatter characteristics. Leukocytes in BAL were identified by CD45 expression. Then the inflammatory cells were identified as follows: alveolar macrophages (Siglec-F+ F4/80+), eosinophils (Siglec-F+ Ly6G-), and neutrophils (CD11b+ Ly6G+).

Supplementary Table S1. Some of the characteristics of the serum donors used in the passive basophil activation test.

	Sex	Age	Group	Blo t 2 sIgE
C1	F	8	Control	0,127
C2	F	9	Control	0,129
C3	M	13	Control	0,214
A1	F	50	Asthma	0,204
A2	F	20	Asthma	0,459
A3	M	19	Asthma	0,646
A4	F	10	Asthma	0,207

Supplementary Table S2. Surface markers used for the flow cytometry analysis to identify the different inflammatory cells in BAL samples.

MARKER	FLUOROCHROME	COMPANY	REFERENCE	Eosinophils	Neutrophils	Alveolar macrophages
CD45	V450	BD	560697	+	+	+
CD3e	PECy7	eBioscience	25-0031-82	-	-	-
CD11b	PE	eBioscience	12-0112-81	+	+	-
CD170 (Siglec-F)	PerCP	eBioscience	46-1702-82	+	-	+
F4/80	FITC	eBioscience	11-4801-81	-	-	+
Ly6G	APC	eBioscience	17-5931-81	-	+	-
